# Incarcerated gallbladder in inguinal hernia: a case report and literature review

**DOI:** 10.1186/s12876-020-01569-5

**Published:** 2020-12-14

**Authors:** János Tajti, József Pieler, Szabolcs Ábrahám, Zsolt Simonka, Attila Paszt, György Lázár

**Affiliations:** Department of Surgery, University of Szeged, Semmelweis u. 8., Szeged, 6725 Hungary

**Keywords:** Incarcerated gallbladder, Hernia, Gallbladder strangulation, Case report

## Abstract

**Background:**

Treating hernias is one of the oldest challenges in surgery. The gallbladder as content in the case of abdominal hernias has only been reported in a few cases in the current literature. Cholecyst has only been described in the content of an inguinofemoral hernia in one case to date.

**Case presentation:**

A 73-year-old female patient was admitted to the Emergency Department due to complaints in the right inguinal area, which had started 1 day earlier. The patient complained of cramp-like abdominal pain and nausea. Physical examination confirmed an apple-sized, irreducible hernia in the right inguinal region. Abdominal ultrasound confirmed an oedematous intestinal loop in a 70-mm-long hernial sac, with no circulation detected. Abdominal X-ray showed no signs of passage disorder. White blood cell count and C-reactive protein level were elevated, and hepatic enzymes were normal in the laboratory findings. Exploration was performed via an inguinal incision on the right side, an uncertain cystic structure was found in the hernial sac, and several small abnormal masses were palpated there. The abdominal cavity was explored from the middle midline laparotomy. During the exploration, the content of the hernial sac was found to be the fundus of the significantly ptotic, large gallbladder. Cholecystectomy and Bassini’s repair of the inguinal hernia were performed safely.

**Conclusions:**

Following a review of the literature, it can be concluded that the finding of incarcerated gallbladder in the content of an inguinal hernia is a rare finding. No other similar emergency case and successful surgical intervention have been reported before.

## Background

Treating hernias is a routine and common surgical intervention and one of the oldest surgical challenges in the world.

The majority, 80%, of hernias of the abdominal wall are inguinal hernias [1]. The most common hernial contents are the following: the greater omentum and certain intestinal segments (the small bowel, appendix, colon, urinary bladder, ovary and fallopian tube). In addition to surgical methods that have been modified several times over time, a number of rare hernial contents have been described; a Meckel’s diverticulum [2] found by Alexandre Littré and an inflamed appendix described by Claudius Amyand [3] should be emphasised from the literature. However, a gallbladder as content in the case of inguinal hernias has only been reported in one case in the current literature [4]. After reviewing the literature, we have concluded that an incarcerated gallbladder in the content of an inguinal hernia is a rare finding, with no other similar cases reported before to the best of the authors’ knowledge.

Our case report summarizes the diagnosis, surgical treatment and perioperative care of this rare case in relation to a review of the current literature.

## Case presentation

In our case report, a 73-year-old female patient was admitted to the Emergency Department for complaints in the right inguinal area, which had started one day earlier. The patient complained of cramp-like abdominal pain and nausea. Bowel movements were normal, and the patient had no fever. From the patient's medical history we should highlight that a right inguinal hernia had been diagnosed years before. At that time surgical consultation had also been involved. However, due to the patient’s severe cardiovascular co-morbidities (aortic, mitral and tricuspid valve insufficiency, right heart failure and low ejection fraction of 22%), the cardiology and preanesthesia consultations determined elective surgical intervention to be very high-risk and the elective operation was therefore contraindicated. In addition the patient had hysterectomy, appendectomy, Alzheimer’s disease and cholelithiasis in her medical history.

**Fig. 1:**
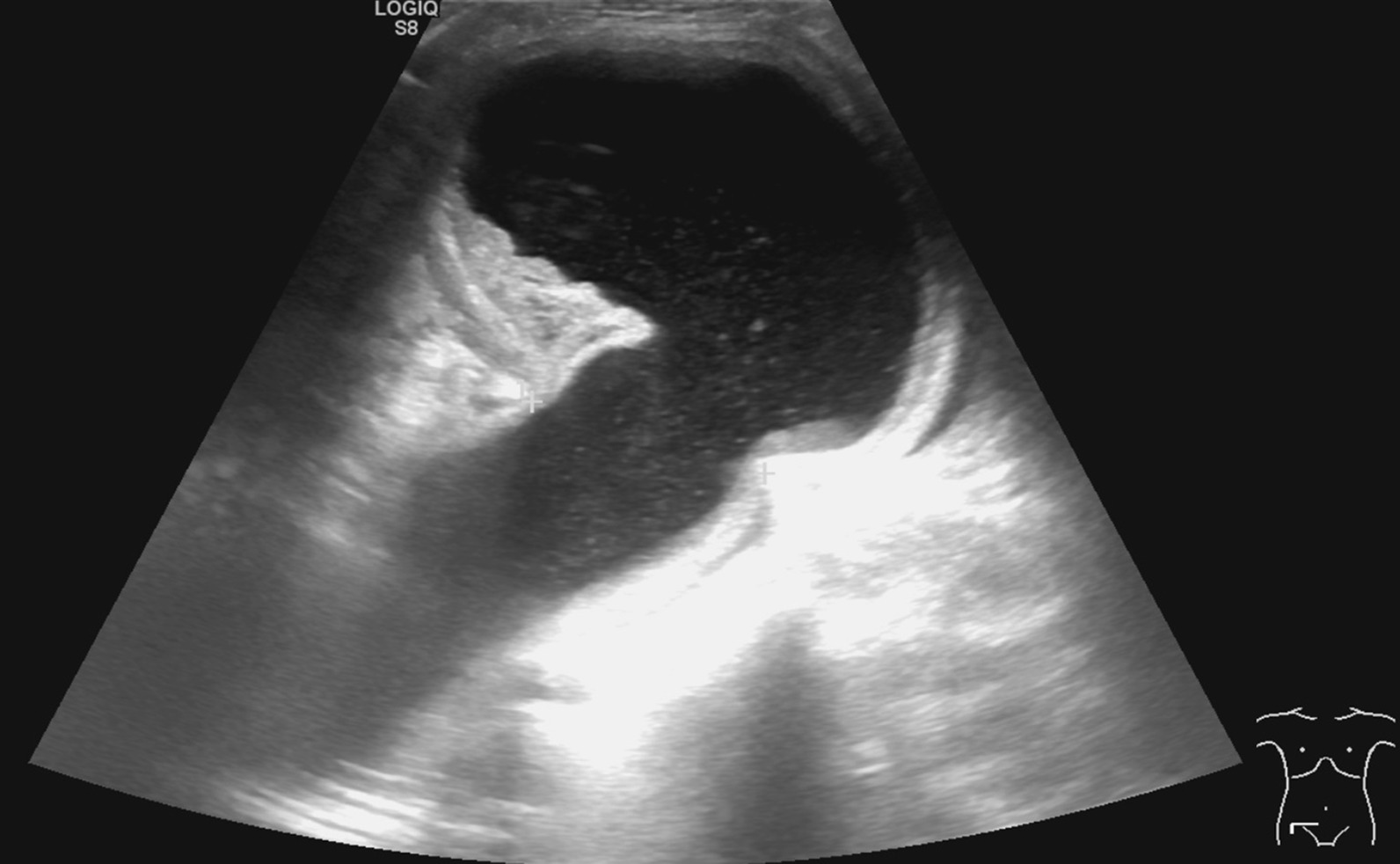
Preoperative abdominal ultrasound showed an oedematous intestinal loop in a 70-mm-long hernial sac protruding via a 30-mm hernial orifice

The physical examination confirmed an apple-sized, irreducible hernia with tenderness to abdominal palpation in the right inguinal region. Skin redness was not detected. Scars from the midline laparotomy and McBurney’s incision were observed. Abdominal ultrasound confirmed an oedematous intestinal loop in a 70-mm-long hernial sac protruding via a 30-mm hernial orifice in the projection of the palpated lesion (Fig. 1). Fluid was seen in the intestinal loop, with no circulation detected there. Neither free abdominal fluid nor a dilated biliary duct was found. An abdominal X-ray showed no signs of intestinal obstruction. White blood cell count was 13.62 G/l, C-reactive protein was 26.6 mg/l, and hepatic enzymes were normal in the patient’s laboratory findings. The physical and ultrasound examinations unequivocally confirmed an incarcerated right inguinal hernia, which was sufficient for a vital indication of acute surgical intervention considering the very high cardiovascular risks.

**Fig. 2:**
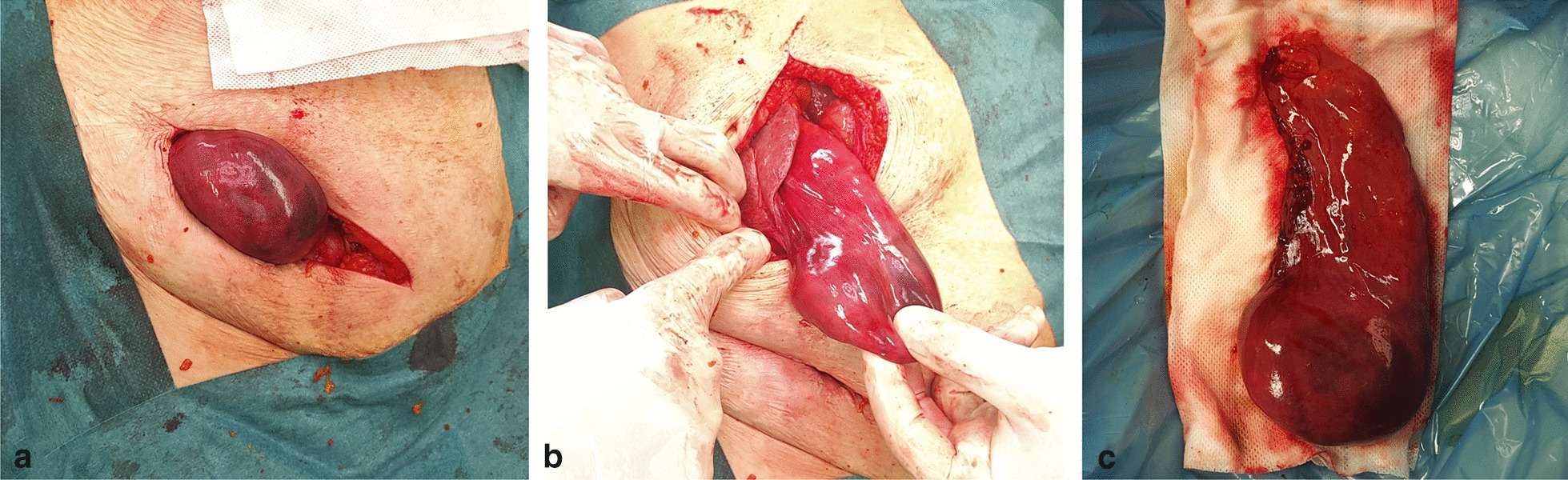
Intraoperative findings. **a** The incarcerated gallbladder detected during inguinal exploration. **b** Ptotic gallbladder elevated from median laparotomy. **c** Specimen

Exploration was performed via an inguinal incision on the right side. In contrast to the abdominal ultrasound finding, an uncertain cystic structure was found in the hernial sac and several small, solid, abnormal masses were palpated in this structure (Fig. 2a). The lesion seemed to be a gynaecological lesion, not an intestinal loop. As regards the uncertain, bizarre finding, the abdominal cavity was explored from the middle midline laparotomy. During the exploration, the content of the hernial sac was found to be the fundus of the significantly ptotic, large gallbladder containing cube-shaped stones palpated earlier during the inguinal exploration. An intramural haemorrhage was observed in this region of the gallbladder, but no clear necrosis was detected (Fig. 2b). There were no signs of inflammation in the hilum of the gallbladder during the surgery. Therefore, a retrograde cholecystectomy and Bassini’s repair of the inguinal hernia were performed safely, and according to our Department protocol a fine silicone drain was left in the omental foramen of Winslow.

Cephalosporin and metronidazole antibiotic therapy and prophylaxis of thrombosis with low molecular weight heparin were administered during the perioperative period. Temporary confusion developed during the postoperative period due to the patient’s Alzheimer’s disease and the stress of the acute surgery; however, no surgical or other organic lesion was found in the background. The drain was removed on the first postoperative day, and oral nutrition was started gradually. Follow-up laboratory findings showed no significant abnormalities. The patient was discharged on the fourth postoperative day, with no signs of inflammation in the wounds, normal passage and no complaints. During the nine-month-long follow-up visits, the sutures were removed and the patient had no significant complaints. Recurrent hernia was not detected. Histology of the gallbladder showed acute cholecystitis with incipient haemorrhagic necrosis in the 210-mm-long sample (Fig. 2c).

## Discussion and conclusions

Incarcerated inguinal hernias are among one of the most common emergency problems in general surgery. Incarcerated hernias represent about 5–15% of operated inguinal hernias [5, 6] and 1.7% of all operated hernias [7]. However, the gallbladder appearing as hernial content in the inguinal region is extraordinary. It is possible that features characteristic of elderly people are responsible for this rare condition, such as a decreased amount of adipose tissue, connective tissue transformation, and the ptotic nature of the gallbladder as a result of the elongation of the peritoneal lining [8]. Based on the literature on the herniation of the gallbladder in PubMed, we can conclude that the mean age of the patients was high (between 40 and 96 years) and the majority of the patients were female [4, 9–18], as in our case.

Until now, only Rodrigues et al. published a case report where the gallbladder had been described in the content of an inguinofemoral hernia [4]. The gallbladder was detected in the femoral canal of an elderly female patient with inguinal complaints. Repair of the abdominal wall and cholecystectomy were performed electively in this case [4]. In most patients, the gallbladder was found in parastomal hernias [9–13], with strangulation of the hernia leading to cholecystitis; there was only one case in which no inflammation was observed. Cholecystectomy was performed in all cases, except for a case report by Frankl et al., where successful conservative therapy was administered after repositioning a hernial sac around a transversostoma in an elderly patient in a poor general condition with polymorbidities [9]. In some cases, the gallbladder was described in hernias developing in the epigastric region or in surgical scars [14–16].

In the majority of the cases, the surgical method used was laparotomy. Only Paolino et al. and Trotta et al. reported cases of laparoscopies, and, in their cases, the gallbladder was found in the content of hernias of the anterior abdominal wall. In the former study, it was combined with Mirizzi’s syndrome [17, 18]. Our team did not opt for laparoscopy due to the cardiac status of the patient. A review of previous cases showed that the proper diagnosis was supported by the abdominal computed tomography in the majority of the cases as well. However, this was not required for our patient due to the clear clinical status, although the intestinal loop described by the abdominal ultrasound as the hernial content was not confirmed by the surgery. In certain publications, hernial repair with mesh is reported during elective surgeries; in our case, this technique could not be used due to the high risk of the incarcerated hernia.

After reviewing the literature, it can be concluded that an incarcerated gallbladder in the content of an inguinal hernia is a rare finding, with no other similar case reported beforehand to the best of the authors’ knowledge. The value of our finding is increased by the fact that a curative surgical intervention was successfully performed on our patient despite her multiple co-morbidities.

## Availability of data and material

Data sharing is not applicable to this article as no datasets have been generated or analysed during the current study.

## References

[1] Bendavid R, Abrahamson J, Arregui ME, Flament JB, Phillips EH (2001). Abdominal wall hernias: principles and management.

[2] Schizas D, Katsaros I, Tsapralis D, Moris D, Michalinos A, Tsilimigras DI (2019). Littre's hernia: a systematic review of the literature. Hernia.

[3] Yagnik VD (2011). Amyand hernia with appendicitis. Clin Pract.

[4] Rodrigues LK, De Jesus RB, Torri GB, Momolli M, Almeida Ghezzi CL, Cavazzola LT (2019). Gallbladder protrusion through the groin region-a very unusual femoral hernia. BJR Case Rep.

[5] Gallegos NC, Dawson J, Jarvis M, Hobsley M (1991). Risk of strangulation in groin hernias. Br J Surg.

[6] Kulah B, Kulacoglu IH, Oruc MT, Duzgun AP, Moran M, Ozmen MM (2001). Presentation and outcome of incarcerated external hernias in adults. Am J Surg.

[7] Romain B, Chemaly R, Meyer N, Brigand C, Steinmetz JP, Rohr S (2012). Prognostic factors of postoperative morbidity and mortality in strangulated groin hernia. Hernia.

[8] McHenry CR, Byrne MP (1986). Gallbladder volvulus in the elderly. An emergent surgical disease. J Am Geriatr Soc.

[9] Frankl J, Michailidou M, Maegawa F (2017). Parastomal gallbladder hernia in a septic patient. Radiol Case Rep.

[10] Garcia RM, Brody F, Miller J, Ponsky TA (2005). Parastomal herniation of the gallbladder. Hernia.

[11] Rosenblum JK, Dym RJ, Sas N, Rozenblit AM (2013). Gallbladder torsion resulting in gangrenous cholecystitis within a parastomal hernia: findings on unenhanced CT. J Radiol Case Rep.

[12] St Peter SD, Heppell J (2005). Surgical images: soft tissue. Incarcerated gallbladder in a parastomal hernia. Can J Surg..

[13] To H, Brough S, Pande G (2015). Case report and operative management of gallbladder herniation. BMC Surg.

[14] Benzoni C, Benini B, Pirozzi C (2004). Gallbladder strangulation within an incisional hernia. Hernia.

[15] Goldman G, Rafael AJ, Hanoch K (1985). Acute acalculous cholecystitis due to an incarcerated epigastric hernia. Postgrad Med J.

[16] Sirikci A, Bayram M, Kervancioglu R (2002). Incisional hernia of a normal gallbladder: sonographic and CT demonstration. Eur J Radiol.

[17] Paolino L, Millan M, Bossi M, Champault G, Barrat C (2011). Herniation of the gallbladder within a hernia of the abdominal wall associated with Mirizzi Syndrome. J Surg Case Rep.

[18] Trotta M, Cesaretti M, Minetti GA, Borgonovo G (2013). Complication of gallbladder herniation through the abdominal wall. Surgery.

